# SEM BSE 3D Image Analysis of Human Incus Bone Affected by Cholesteatoma Ascribes to Osteoclasts the Bone Erosion and VpSEM dEDX Analysis Reveals New Bone Formation

**DOI:** 10.1155/2020/9371516

**Published:** 2020-02-15

**Authors:** Michela Relucenti, Selenia Miglietta, Gabriele Bove, Orlando Donfrancesco, Ezio Battaglione, Pietro Familiari, Claudio Barbaranelli, Edoardo Covelli, Maurizio Barbara, Giuseppe Familiari

**Affiliations:** ^1^Department SAIMLAL Section of Human Anatomy, Laboratory of Electron Microscopy “Pietro M. Motta”, Sapienza University of Rome, Via Alfonso Borelli 50, 00161 Rome, Italy; ^2^Department NESMOS, Neurosurgery Unit, Sapienza University of Rome, Via di Grottarossa 1039, 00189 Rome, Italy; ^3^Department of Psychology, Sapienza University of Rome, Via dei Marsi 78, 00185 Rome, Italy; ^4^Department NESMOS, ENT Unit, Sapienza University of Rome, Via di Grottarossa 1039, 00189 Rome, Italy

## Abstract

Bone erosion is considered a typical characteristic of advanced or complicated cholesteatoma (CHO), although it is still a matter of debate if bone erosion is due to osteoclast action, being the specific literature controversial. The purpose of this study was to apply a novel scanning characterization approach, the BSE 3D image analysis, to study the pathological erosion on the surface of human incus bone involved by CHO, in order to definitely assess the eventual osteoclastic resorptive action. To do this, a comparison of BSE 3D image of resorption lacunae (resorption pits) from osteoporotic human femur neck (indubitably of osteoclastic origin) with that of the incus was performed. Surface parameters (area, mean depth, and volume) were calculated by the software Hitachi MountainsMap© from BSE 3D-reconstructed images; results were then statistically analyzed by SPSS statistical software. Our findings showed that no significant differences exist between the two groups. This quantitative approach implements the morphological characterization, allowing us to state that surface erosion of the incus is due to osteoclast action. Moreover, our observation and processing image workflow are the first in the literature showing the presence not only of bone erosion but also of matrix vesicles releasing their content on collagen bundles and self-immuring osteocytes, all markers of new bone formation on incus bone surface. On the basis of recent literature, it has been hypothesized that inflammatory environment induced by CHO may trigger the osteoclast activity, eliciting bone erosion. The observed new bone formation probably takes place at a slower rate in respect to the normal bone turnover, and the process is uncoupled (as recently demonstrated for several inflammatory diseases that promote bone loss) thus resulting in an overall bone loss. Novel scanning characterization approaches used in this study allowed for the first time the 3D imaging of incus bone erosion and its quantitative measurement, opening a new era of quantitative SEM morphology.

## 1. Introduction

Consensus-based recommendations for the definition of advanced or complicated cholesteatoma (CHO) [1] state that it is an agglomerate of keratinizing squamous epithelium, subepithelial connective tissue, that grows as a progressive accumulation of keratin debris with/without surrounding inflammatory reaction. Regarding its microstructure CHO is made of matrix (keratinized squamous epithelium), perimatrix (subepithelial connective tissue of variable thickness), and keratin debris. Bone erosion is considered a typical characteristic of cholesteatoma; however, it is still a matter of debate if bone erosion is due to osteoclast action, being present in literature conflicting results [2–7]. Scanning electron microscopy is an elective imaging technique for bone ultrastructural studies [8–12], so we observed by means of innovative SEM BSE 3D imaging and VpSEM EDX analysis that cholesteatoma affected incus bone surface, in order to accurately describe their surface modifications and finally assess if osteoclasts are directly responsible for bone resorption. To accomplish this task, we compared, using SEM BSE 3D imaging analyzed by Hitachi MountainsMap software, the fine structure of resorption pits observed on incus bone surface with the resorption lacunae from osteoporotic femur neck, indubitably of osteoclastic origin. Ultrastructural topography of incus bone surface was also studied through VpSEM EDX analysis.

## 2. Materials and Methods

### 2.1. Samples

We observed eighteen incus bones recovered during surgical procedures of CHO removal obtained with patients' informed consent and 1 unaffected incus bone (the control) from cadaver.

We studied eighteen femoral neck biopsies from postmenopausal women with hip arthrosis and osteoporosis who underwent surgical hip substitution, 1 femoral neck biopsy from woman without osteoporosis. BMD and T-score to assess bone osteoporosis condition were evaluated by DEXA (Hologic Delphi) before the surgical operation. Samples were obtained with patients' informed consent.

The study was approved by the Institutional Ethics Board and adhered to the tenets of the Declaration of Helsinki.

### 2.2. SEM Protocols

#### 2.2.1. Femoral Neck Biopsies

Samples were fixed immediately upon recovery in 2.5% glutaraldehyde in PBS at 4°C for 48 h, then immersed in a 3% hydrogen peroxide solution for 48 h at room temperature (for bone marrow removal), and then rinsed with distilled water. Samples were then sonicated in a sonic device [13] in distilled water at room temperature, rinsed with distilled water, and dehydrated in acetone series. Samples were finally dried using a critical point dryer (Emitech K850, Emitech, Corato, Italy), mounted on aluminum stubs, platinum coated using an Emitech K 550 sputter coater (Emitech, Corato, Italy), and observed by a Hitachi FE SEM S 4000 operating at 7 kV. SEM micrographs were acquired with a DISS5 Digital Image Scanning System (point electronic, Germany).

#### 2.2.2. Incus Preparation Protocol for SEM

Samples were fixed immediately upon recovery in 2.5% glutaraldehyde in PBS at 4°C for 48 h; then, they were gently sonicated in an ultrasonic device (to remove excess of keratinizing squamous epithelium that would have prevented surface observation). Fifteen samples were prepared for SEM (as previously described for femur neck) and sputter coated with platinum using an Emitech K 550 sputter coater (Emitech, Corato, Italy). Observations were conducted by a Hitachi FE SEM S 4000 operating at 7 kV and by a Hitachi SU 3500 (Hitachi High-Technologies Europe GmbH, Mannheim, Germany), at 10 kV in SE mode.

#### 2.2.3. Incus Preparation Protocol for VpSEM and EDX Microanalysis

Three samples, after fixation in 2.5% glutaraldehyde in PBS at 4°C for 48 h, were only gently sonicated in a sonic device [13] and then directly observed by a Hitachi SU 3500 (Hitachi High-Technologies Europe GmbH, Mannheim, Germany), operating at 5 kV and 60 Pa, in BSE COMPO mode without metal coating.

### 2.3. BSE 3D Image Analysis

Hitachi SU 3500 is equipped with a four-quadrant BSE detector that allows to acquire four images simultaneously with only one scan. The four pictures are then integrated into 3D images and processed to extract quantitative information (all those steps were performed by the software Hitachi Map 3D 7.4 Digital surf, Besançon, France). To obtain this kind of data is extremely useful to implement the morphological classification parameters usually used to characterize resorbing and forming bone surfaces. In fact, acquisition of quantitative resorption pit information such as area, mean depth, and volume allows to compare pits from different sources (femur and incus) and finally assess if they have the same origin. Regions containing resorption bay were analyzed in both incus bone and femur neck samples. BSE 3D images of well delimited resorption pits were acquired, 4 images were combined by the software, and 3D reconstruction was obtained. Resorption pit area, mean depth, and volume were extracted by MountainsMap software after 3D image reconstruction. In more detail, we performed single pit selection on the 3D image reconstruction, followed by automatic measurement of area, mean depth, and volume. Data were collected and statistically analyzed by SPSS statistical software. The following test was performed: summary statistic to assess the normality of distribution of pit area, mean depth, and volume values; independent sample *t*-test (assuming unequal and equal variances) was used to compare pits area, mean depth, and volume values between incus and femur samples.

### 2.4. EDX Microanalysis

The variable pressure scanning electron microscopy used in this study (VP-SEM, Hitachi SU3500) is equipped with dual energy dispersive X-ray spectroscopy (dEDS, Bruker XFlash® 6|60) detectors. This instrument has the ability to perform simultaneously multimodal imaging and spatial distribution chemical mapping, a truly powerful analytical approach to study biological surfaces in their native state. The XFlash® 6|60 is particularly suitable for applications with relatively low X-ray yield, as common in the area of nanoanalysis.

### 2.5. Morphological Classification Parameters for Bone Surface Evaluation

Incus bone areas were classified as resorptive and forming bone surfaces, according to widely accepted morphological criteria described in literature [8–12, 14–17]. Briefly, resorbing bone surfaces are characterized by the presence of large resorption bay or scattered resorption pits (Howship's lacunae). Those structures observed by SEM show shining bright rounded edges, a floor made of partially demineralized collagen bundles and punctuated by narrow gutters, that appear darker at BSE imaging mode. Bone forming surfaces are characterized by an irregular surface, with collagen bundles undergoing mineralization, mineralizing matrix vesicles and shallow pits (the osteocytic lacunae) in which osteoblast/osteocyte immure themselves. They have an irregular ellipsoidal shape with a large range of variation [18].

## 3. Results and Discussion

Each CHO incus sample was observed by SEM at low magnification following a precise scanning pathway, in order to assess the general bone morphology and define areas suitable for high magnification observations. This method allowed counting of nutrient foramina opening onto the surface (49 foramina on 18 bones) and identification of areas with marked bone erosion and, interestingly, areas with new bone formation. It is still a matter of debate if bone erosion is due to osteoclast action; moreover, new bone formation was never been described in the incus bone affected by CHO. To get an insight on these findings, we performed observations at magnifications ranging from 400x to 600x, 3D image reconstruction, and EDS analysis.

### 3.1. Observations of Normal Sample Surface

**Figure 1:**
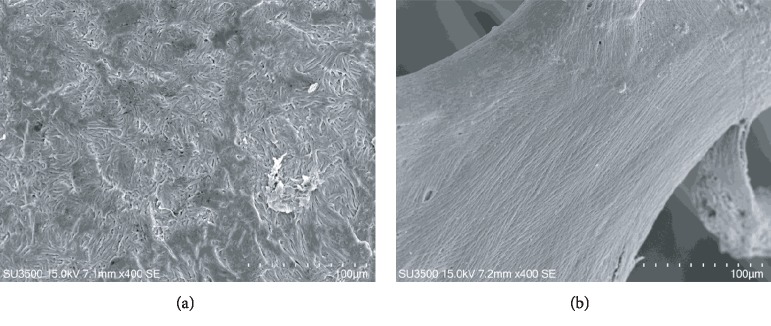
(a) SE mode, 400x. Incus bone surface from cadaver, normal surface. (b) SE mode, 400x. Trabecular bone from patient without osteoporosis, normal surface.

Before showing images of samples with resorption areas, two images of normal surfaces are presented (Figure 1): normal incus bone surface (Figure 1(a)) and normal trabecular bone (Figure 1(b)). The surface of both bones is devoid of resorption bays.

### 3.2. Observations of Resorbing Areas

**Figure 2:**
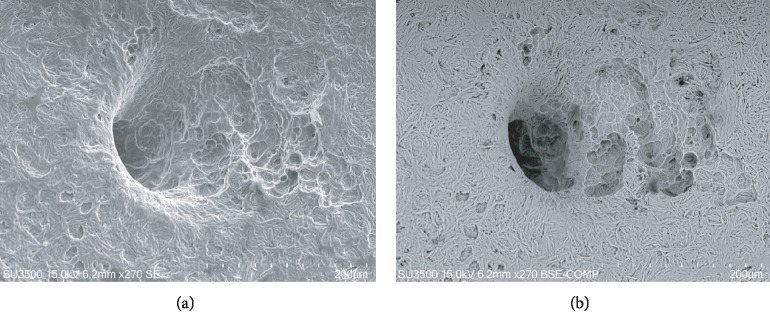
(a) SE mode, 270x. Nutritive foramen from CHO incus bone. On the right side of the image, large resorption bays, extending since into the foramen, are visible. On the left corner of the picture, osteocytic lacunae are visible. (b) BSE-COMP mode, 270x of same sample. Darker (demineralized) areas correspond to deeper resorption bays. This field shows both bone resorption and bone formation phenomena.

Images of CHO incus bone surface showed 67% of nutritive foramina surrounded by large resorption bays that seem to radiate from nutritive foramen opening (Figures 2(a) and 2(b)).

**Figure 3:**
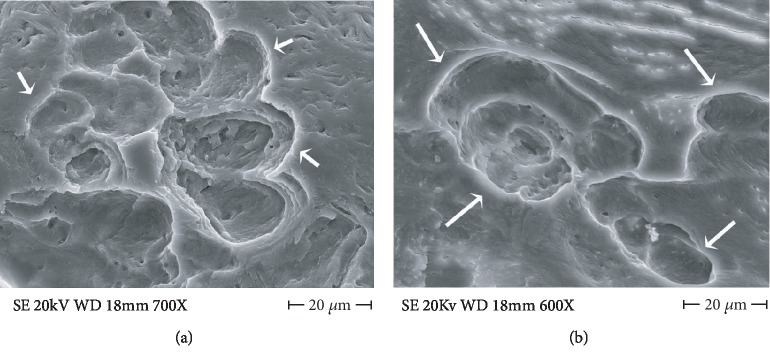
(a) FE SEM 700x, CHO incus bone resorption bay at higher magnification, osteoclast snake trail pathway is visible (arrows). At the center of the resorption bay, a small promontory rises being relatively resistant to resorption. (b) FE SEM, 600x, osteoclastic resorption bay on osteoporotic human femur neck (arrows), they are unequivocally of osteoclast origin and are undistinguishable from those in (a).

Observed at higher magnification CHO incus bone resorption bays and pits (Figure 3(a)) resemble in all respects those on the surface of femur neck with osteoporosis (Figure 3(b)).

**Figure 4:**
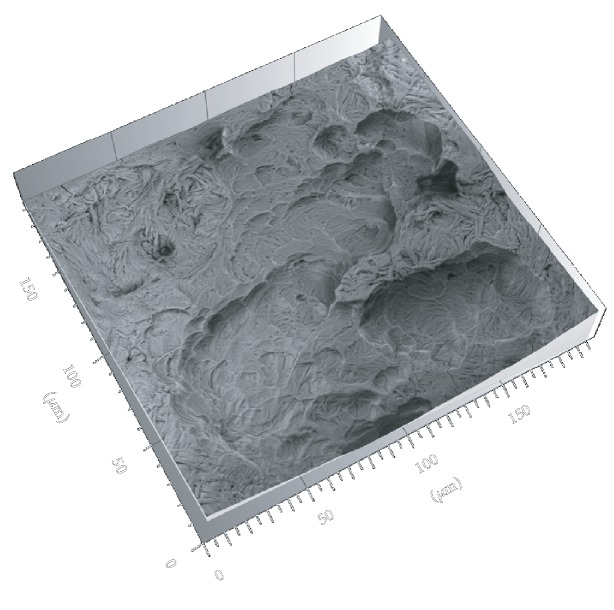
3D reconstruction from 4 images in BSE mode. Each resorption bay contains several pits.

To definitely assess if incus bone resorption bay is a product of osteoclasts action, we used Hitachi MountainsMap© software to perform a 3D reconstruction from 4 BSE mode images (Figure 4).

**Figure 5:**
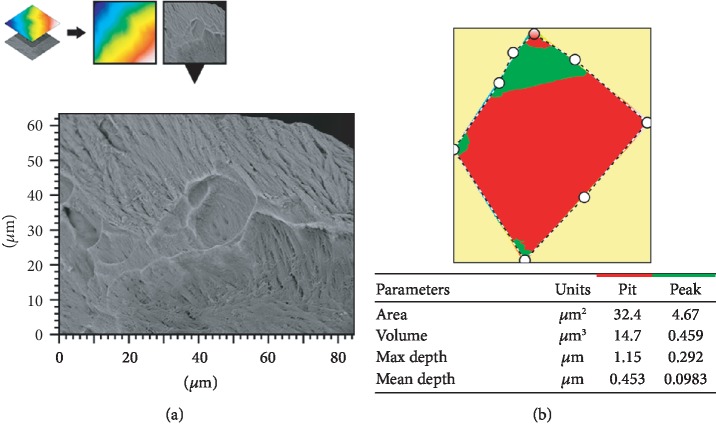
(a) The extracted area of a resorption bay from a larger 3D-reconstructed image. (b) A delimited single pit from which software calculated parameter values.

A small area was extracted from a 3D-reconstructed image, and each single pit in the small area was analyzed by the software to calculate: area, mean depth, and volume (Figures 5(a) and 5(b)).

**Figure 6:**
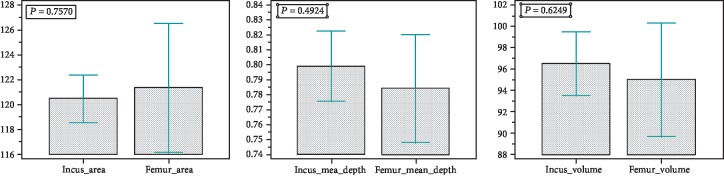
Graphs represent distribution of pit measurement data (from the left to the right): incus area vs. femur area; incus mean depth vs. femur mean depth; incus volume vs. femur volume.

We analyzed 79 pits, for each considered parameter values which were recorded and statistically evaluated by SPSS statistical software. Firstly, a summary statistic was performed on data collected for each parameter, to assess normality of distribution (Table 1). For all values, data distribution was normal, so *t*-test was performed between values of each parameter measured on incus and femur to assess difference between values (Table 2, Figure 6). Two series of independent sample *t*-test, assuming, respectively, unequal and equal variances, were conducted to compare area, mean depth, and volume values between incus and femur. Both series evidenced that no difference exists between the two groups (*p* > 0.05) for each considered parameter.

### 3.3. Observation of New Bone-Forming Area

**Figure 7:**
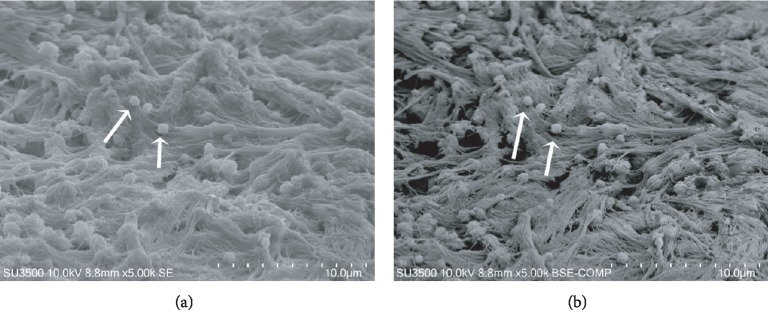
SE, BSE comp 5000x, new bone formation on CHO incus bone surface. (a) SE mineralizing matrix vesicles releasing their content on collagen bundles (arrows). (b) BSE comp mineralizing matrix vesicles (arrows) appear as bright and rough spheres. Collagen fibres and bundles with variable mineralization degree are visible. Mineralized areas appear lighter at BSE mode.

The detailed incus surface observation allowed another interesting finding, the observation of new bone-forming areas on incus surface. Our SEM images are the first to show this process on incus. Mineralizing vesicles releasing their content on collagen bundles are shown in Figures 7(a) and 7(b). SE mode allows the detailed visualization of collagen fibre meshwork while BSE mode clearly points out that matrix vesicles are filled with a high molecular weight content and that collagen bundles have different mineralization degree (lighter or darker areas).

**Figure 8:**
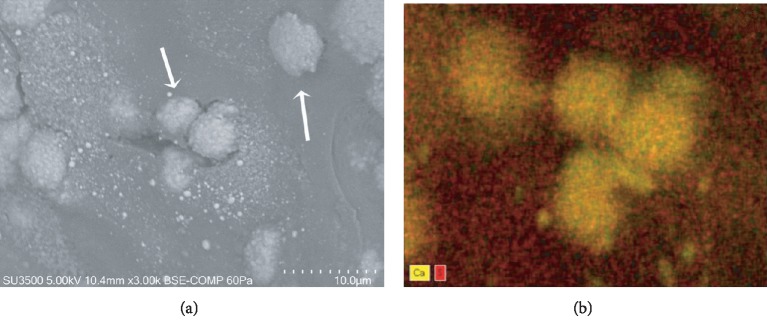
BSE Comp, 3000x, dEDS analysis, confirmation of new bone formation on CHO incus bone. (a) VP SEM BSE image shows matrix vesicles (arrows). (b) Elemental distribution (dEDS analysis) allows identification of chemical species, calcium in matrix vesicles (yellow) and sulphur in extracellular matrix (red).

These areas were also analyzed in uncoated samples by variable pressure SEM dEDS analysis. Variable pressure SEM allows the observation of uncoated samples, avoiding metal coating disturbance during elemental analysis. Areas containing calcified matrix vesicles (Figure 8(a)) were analyzed by dEDS. Elemental mapping (Figure 8(b)) clearly shows the presence of calcium in matrix vesicles, while sulphur, contained in matrix proteoglycans, is present only in the surrounding extracellular matrix. Calcium and phosphorus are the characteristic elements of bioapatite [19–21]. The elemental mapping clearly demonstrates that matrix vesicles have a calcium phosphate content.

**Figure 9:**
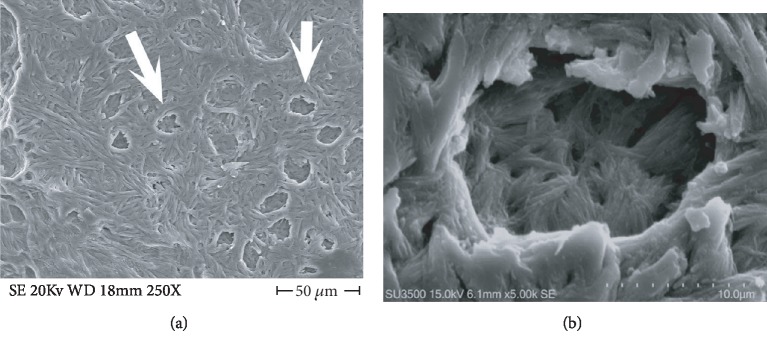
New bone formation on CHO incus bone surface (a), FE SEM, 250x, osteocytic lacunae (arrows) formed by self-immuring osteocytes. (b) SE, 5000x, high magnification of an osteocytic lacuna, the floor appears less mineralized and spotted by deep holes to accommodate osteocyte cellular processes.

A later stage in new bone formation is represented by osteocyte self-immuring in forming bone areas. In Figure 9, detailed images of osteocytic lacunae are presented for the first time in which the osteocyte self immure on incus surface. Here again, they are perfectly superimposable with osteocytic lacunae on femur neck samples. Osteocytic lacunae appear surrounded by fully mineralized collagen bundles. At higher magnification (Figure 8(b)), on the floor of the osteocytic lacuna, the not yet fully mineralized collagen fibres and the deep holes are visible in which osteocyte cellular processes nestle.

Prominent theories on bone resorption in CHO are osteoclast activation; pressure necrosis; and acid lysis, enzyme mediation, and inflammatory mediation [22]. The mechanism of bone erosion in middle ear cholesteatoma remains still unclear, although its histopathology has been intensively studied.

In some studies [2, 3], osteoclast was not observed in resorbing bone areas of incus with CHO; in others [4–7], they were reported. This is probably due to the transient nature of osteoclasts; they have a relatively short life and, being surgical procedures done after inflammation control, they may be not present at time of sample recovery and fixation.

Our results showed that no difference exists between area, mean depth, and volume values between incus and femur resorption pit, allowing us to state that surface erosion on the incus is due to osteoclast action.

**Figure 10:**
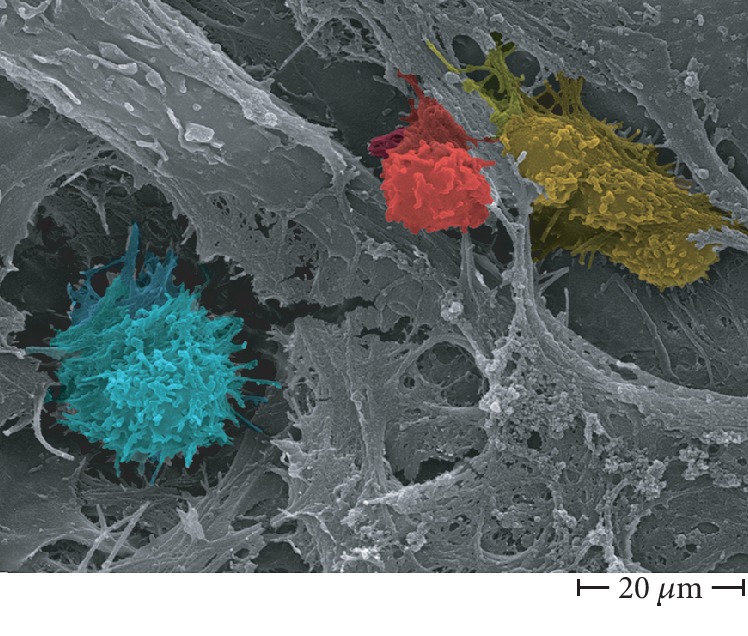
Inflammatory cells and an osteoclast on incus affected by CHO surface, FE SEM 3000x. Active macrophage (blue), lymphocyte (red), and osteoclast (yellow).

Osteoclasts are multinucleated cells, they differentiate from monocyte-lineage hematopoietic precursor cells [23]. Macrophage colony-stimulating factor (M-CSF) and receptor activator of nuclear factor *κ*B ligand (RANKL) regulate both differentiation and activation of osteoclasts [24]. In several inflammatory diseases, like rheumatoid arthritis, pathological bone loss is observed, together with RANKL overproduction [25, 26]. Immune cells such as T-lymphocytes and macrophages that infiltrate into damaged areas are major sources of RANKL [27], and fibroblasts in the cholesteatoma perimatrix express RANKL [28]. The effector cell of focal osteolysis is the osteoclast, but cytokines are key regulators of inflammatory osteolysis [29]. IL-1, IL-6, TNF-*α*, and prostaglandin E2 (PGE2) have been investigated as inflammatory mediators of cholesteatoma progression. They are assumed to enhance bone resorption by activating osteoclasts [30–32], and inflammation has been confirmed to be essential for cholesteatoma formation, growth, and expansion, including the bone resorption process [22, 33, 34]. Inflammatory cells were observed in our samples; in Figure 10, a rare coexistence of a macrophage (blue), a lymphocyte (red), and an osteoclast is presented [35–37].

Bone homeostasis is maintained balancing bone-resorbing osteoclast and bone-forming osteoblast activity, alteration of this balance causes bone loss, that is not recovered by new bone formation. In fact, in inflammation, disease-like RA bone erosion results from excessive bone resorption and markedly limited bone formation [38]. We observed typical morphological markers of new bone formation on incus by CHO, but this phenomenon probably happens at a slower rate than bone resorption, so that bone loss is not compensed.

## 4. Conclusions

The innovative quantitative approach used in this paper implements the classical surface morphological characterization, allowing us to state that surface erosion of the incus is due to osteoclast action. Moreover, our observation and processing image workflow are the first in the literature showing the presence not only of bone erosion but also of matrix vesicles releasing their content on collagen bundles and self-immuring osteocytes, all markers of new bone formation on incus bone surface. On the basis of recent literature [22–34], it has been hypothesized that inflammatory environment induced by CHO may trigger the osteoclast activity, eliciting bone erosion; we can provide a morphological evidence of this hypothesis in Figure 9; in fact, a T-lymphocyte, a macrophage, and an osteoclast were photographed one near the other; the photograph gives the impression of witnessing the paracrine molecular dialogue between these cells [22–34]. The observed new bone formation probably takes place at a slower rate in respect to the normal bone turnover, and the process is uncoupled (as recently demonstrated for several inflammatory diseases that promote bone loss) thus resulting in an overall bone loss. Novel scanning characterization approaches used in this study allowed for the first time the 3D imaging of incus bone erosion and its quantitative measurement, opening a new era of biological quantitative SEM morphology. Taken all together, our morphological results let us hypothesize that cholesteatoma creates an environment of chronic infection with peculiar biochemical characteristics that alters normal bone turnover on incus bone. Targeting the cell population of the inflammatory microenvironment (which produce molecules that stimulate osteoclast activity) will open new therapeutic options, in particular in the field of noninvasive therapies, allowing to inhibit bone erosion development in the acquired middle ear cholesteatoma.

## Figures and Tables

**Table 1:** Summary statistic of area, mean depth, and volume values.

Pit	Distribution	Area *μ*m^2^ Arithmetic mean ± ds	Mean depth *μ*m Arithmetic mean ± ds	Volume *μ*m^3^ Arithmetic mean ± ds
Incus	Normal	120.48 ± 8.54	0.799 ± 0.10	96.48 ± 13.16
Femur neck	Normal	121.34 ± 23.2047	0.784 ± 0.16	94.99 ± 23.65

**Table 2:** Independent sample *t*-test on area, mean depth, and volume values.

	Area		Mean depth		Volume	
	Incus	Femur	Incus	Femur	Incus	Femur
Sample size	79	79	79	79	79	79
Arithmetic mean	120.48	121.34	0.799	0.784	96.48	94.99
95% CI for the mean	118.57 to 122.39	116.15 to 126.54	0.77 to 0.82	0.74 to 0.82	93.51 to 99.45	89.69 to 100.28
Variance	72.95	538.45	0.011	0.025	173.31	559.32
St deviation	8.54	23.20	0.10	0.16	13.16	23.65
St error mean	0.96	2.61	0.011	0.018	1.49	2.66
*F*-test equal variances	*p* < 0.001		*p* < 0.001		*p* < 0.001	
*t*-test equal variances	*t*(156) = 0.310 *p* = 0.7568		*t*(155) = −0.688 *p* = 0.4922		*t*(156) = −0.489 *p* = 0.6258	
Levene *t*-test unequal variances	*t*(98.76) = 0.310 *p* = 0.7570		*t*(122.43) = −0.688 *p* = 0.4924		*t*(134.18) = −0.489 *p* = 0.6249	

## Data Availability

Data are stored in computer of our institution and are available upon request.
